# Risk of Recurrent Bleeding Events in Nonvalvular Atrial Fibrillation Treated with Vitamin K Antagonists: A Clinical Practice Research Datalink Study

**DOI:** 10.1055/s-0039-1698413

**Published:** 2019-10-04

**Authors:** Raza Alikhan, Cinira Lefevre, Ian Menown, Steven Lister, Alex Bird, Min You, David Evans, Cormac Sammon

**Affiliations:** 1 Haemophilia and Thrombosis Centre, University Hospital of Wales, Cardiff, United Kingdom; 2 Center of Observational Research and Data Sciences, Bristol-Myers Squibb, Rueil-Malmaison, France; 3 Craigavon Cardiac Centre, Craigavon, United Kingdom; 4 UK Health Economics and Outcomes Research, Bristol-Myers Squibb, Uxbridge, United Kingdom; 5 Health Economics and Outcomes Research, Pfizer, Surrey, United Kingdom; 6 Center of Observational Research and Data Sciences, Bristol-Myers Squibb, Plainsboro, New Jersey, United States; 7 PHMR Ltd., London, United Kingdom

**Keywords:** VKA, bleeding, atrial fibrillation, risk

## Abstract

**Background** There is little evidence on how the occurrence of a bleed in individuals on vitamin K antagonists (VKAs) impacts the risk of subsequent bleeds, and thromboembolic and ischemic events. Such information would help to inform treatment decisions following bleeds.

**Objective** To estimate the impact of bleeding events on the risk of subsequent bleeds, venous thromboembolism (VTE), stroke, and myocardial infarction (MI) among patients initiating VKA treatment for new-onset nonvalvular atrial fibrillation (NVAF).

**Methods** We conducted an observational cohort study using a linked Clinical Practice Research Datalink—Hospital Episode Statistics dataset. Among a cohort of individuals with NVAF, the risk of clinically relevant bleeding, VTE, stroke, and MI was compared between the period prior to the first bleed and the periods following each subsequent bleed. The rate and cost of general practitioner (GP) consultations, prescriptions, and hospitalizations were also compared across these periods.

**Results** The risk of clinically relevant bleeding events was observed to be elevated at least twofold in all periods following the first bleeding event. The risk of VTE, stroke, and MI was not found to differ according to the number of clinically relevant bleeding events. The rate and cost of GP consultations, GP prescriptions, and hospitalizations were increased in all periods relative to the period prior to the first bleed.

**Conclusions** The doubling in the risk of bleeding following the first bleed, taken alongside the stable risk of MI, VTE, and stroke, suggests that the risk–benefit balance for VKA treatment should be reconsidered following the first clinically relevant bleed.

## Introduction

Atrial fibrillation (AF) is the most common of the cardiac arrhythmias, affecting more than 4.5 million people in the European Union, and is responsible for approximately one-third of cardiac rhythm hospitalizations. 1 2 From the 1980s to the early 2000s, there has been a 60% increase in hospital admissions as a result of AF, 3 4 5 and it is estimated that there will be a threefold increase in the prevalence of AF over the next 50 years. 3 6

AF is associated with an increased risk of arterial thromboembolism and ischemic stroke, and the use of oral anticoagulant (OAC) treatments is therefore recommended for patients with AF and elevated CHA2DS2-VASc (stroke) risk scores. 7 8 Conventionally, vitamin K antagonists (VKAs) have been used for anticoagulation for stroke prevention in AF patients as well as the treatment and prevention of recurrent venous thromboembolism (VTE). Although VKAs appear to be effective in stroke prevention when optimally dosed, they are associated with an increase in the risk of bleeding. 9 10 Non-VKA OACs (NOACs), a class of alternative anticoagulant treatment options, have been found to be associated with a 50% lower risk of intracranial hemorrhage than VKAs. 11 NOACs are therefore increasingly used as an alternative treatment option when initiating OAC treatment. 12 13

Studies that have quantified the relationship between anticoagulant use and the risk of bleeds in AF patients have generally considered the first bleed only or assumed that having a bleed does not alter the risk of experiencing subsequent bleeds. However, a bleed is a significant clinical event which may alter the risk of both subsequent bleeds and/or other clinical events. As any change in risk following a bleed may impact the balance of the risks and benefits of treatment, there is a need for evidence to support treatment decisions in this setting. 7 14

The aim of this study was to estimate the impact of bleeding events on the risk of subsequent bleeds and other clinical outcomes (VTE, stroke, and myocardial infarction [MI]) among patients initiating VKA treatment for new-onset nonvalvular AF (NVAF) and to explore whether there is an incremental change in risk with increasing number of bleeds. The impact of bleeding events on the rate of health care resource utilization (HCRU) and health care costs in NVAF patients initiating VKA was also investigated.

## Methods

### Source of Data

The study was performed using a linked Clinical Practice Research Datalink (CPRD)—Hospital Episode Statistics (HES) dataset. This dataset combines anonymized medical-record data for patients registered with participating general practitioners (GPs) in England with details of their admissions to National Health Service (NHS) hospitals. The linked database therefore includes longitudinal information on diagnoses, symptoms, laboratory tests, and prescriptions issued by the GP in addition to information on referrals to specialist, hospital discharge diagnoses, hospital procedures, and deaths. 15 The database has been used extensively in pharmacoepidemiological research and the recording of diagnoses has been found to have high validity. 16

### Study Population

The study population consisted of all individuals in the CPRD-HES linked database with a VKA prescription recorded between the January 1, 2003 and the December 31, 2013 who met the following criteria: AF diagnosis recorded prior to their first VKA prescription, no evidence of valvular AF, at least 18 years old at first VKA prescription, at least 12 months of follow-up prior to the first VKA prescription, no evidence of prior NOAC of VKA use, and registered with a practice meeting CPRD quality criteria for at least 12 months before the first VKA prescription, with records that met CPRD quality criteria ( Supplementary Fig. S1 ).

**Fig. 1:**
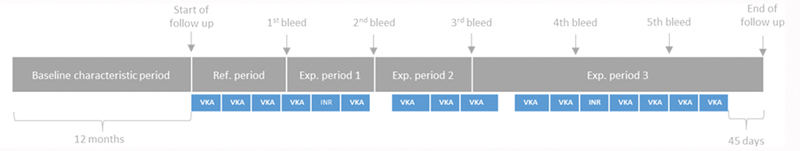
Schematic description of study design. Individuals are followed up from their first VKA prescription until discontinuation of VKA treatment (unless the study period ends, the individual leaves the database, or the individual dies before discontinuing treatment; not shown). In the analysis the risk of primary and secondary outcomes within each of exposure periods 1 to 3 (defined by the number of bleeds that has occurred; 1, 2, or ≥3) was compared with the risk in the reference period (the period prior to the first bleed under VKA treatment). Adjusted models include covariate information extracted from the baseline characteristic period (12-month period prior to the first VKA prescription). VKA, vitamin K antagonist.

Individuals were followed up for the duration of their VKA treatment such that the start of follow-up for each individual in the population was defined as the date of their first VKA prescription (index date) and the end of follow-up was defined as the earliest of either the date they discontinued VKA treatment, December 31, 2012 (end of study period), or the date they left the database or death. Individuals were considered to have discontinued VKA treatment following their first 45-day gap in prescribing ( Fig. 1 ); a 45-day gap was used to define discontinuation based on the assumption that a prescription may last as much as 30 days longer than expected based on dose modifications and that the five-half-life elimination period of warfarin from the body is approximately 15 days. Records of international normalized ratio (INR) measures were used as a proxy of a VKA prescription with a duration of 45 days in an effort to account for VKA prescriptions issued outside the GP practice.

### Study Outcomes

The primary outcome variable was any bleed (AB) event, defined as a composite of major bleed (MB) and clinically relevant nonmajor bleed (CRNMB) events. Consistent with International Society on Thrombosis and Haemostasis definitions, 17 MB was defined as any bleeding event which occurred in a critical area or organ (i.e., intracranial, intraspinal, intraocular, pericardial, intra-articular, intramuscular, and retroperitoneal), which was associated with a record of ≥2 g/dL drop in hemoglobin, and occurred within 10 days of a record for a blood transfusion or within 10 days of death. A bleeding event was defined as CRNMB if it did not meet the criteria for MB but led to a hospital admission (i.e., was recorded in the HES database), led to medical or surgical treatment, or led to discontinuation of VKA treatment. MB, CRNMB, and intracranial bleeds (ICB) were included as secondary outcomes in addition to MI, VTE, and ischemic and hemorrhagic stroke. MB and CRNMB were identified as outlined above, while the other outcomes were identified using relevant Read and International Classification of Diseases 10th Revision (ICD-10) codes.

### Covariates

Age, gender, ethnicity, region of residence, body mass index (BMI), smoking status, and alcohol consumption at the time of the first VKA prescription were extracted for each individual where available. Records indicative of hypertension, diabetes mellitus, congestive heart failure, peripheral artery disease, liver disease, and malignancies recorded in the 12 months preceding the first VKA prescription were also extracted. CHA2DS2-VASc scores at index date were calculated as the sum score for age (+1 if patient was 65–74 years old, +2 if patient ≥75 years), congestive heart failure history (+1 if yes), hypertension history (+1 if yes), stroke/transient ischemic attack/thromboembolism history (+2 if any is yes), vascular disease history (+1 if yes), diabetes mellitus (+1 if yes), and gender (+1 if female). HAS-BLED scores at index date were calculated as previously described 7 by combining data on hypertension, abnormal renal/liver function, stroke/thromboembolism, bleeding history, age >65 years, and drug consumption/alcohol abuse. Labile INR is also a component of the HAS-BLED score but was not included owing to incomplete INR reporting in the CPRD. The HAS-BLED score in our study, therefore, ranged from 0 to 8, as in other database studies. 18 19 Information on the concomitant prescribing of aspirin, nonsteroidal antiinflammatory drugs (NSAIDs; diclofenac, ibuprofen, and naproxen), cyclooxygenase (COX) inhibitors, and antiplatelet agents (clopidogrel, prasugrel, and ticagrelor) was also extracted.

### Statistical Analysis

Baseline characteristics were described for all individuals in the study population and by the total number of bleeds an individual had during follow-up. Time-varying Cox models, with “number of bleeds” included as a four-level time-varying categorical variable, were used to estimate hazard ratios (HRs) comparing the hazard of each outcome following the first, second, and third bleeds to the hazard of that outcome in the period prior to the first bleed. As the number of bleeds was coded in a time-varying manner, a single individual could potentially contribute to all four exposure periods ( Fig. 1 ). Unadjusted and adjusted HRs with 95% confidence intervals (CIs) were estimated with the adjusted results accounting for the following baseline patient characteristics: age, gender, region of residence, BMI, smoking, alcoholism, hypertension, diabetes mellitus, congestive heart failure, peripheral artery disease, liver disease, CHA2DS2-VASc score, HAS-BLED score, and aspirin use. Collinearity among the clinical risk scores and the individual covariates they are composed of was tested for, where present the individual covariates were excluded from the analysis.

To estimate the impact of incremental bleeding events on HCRU, we used log-Poisson generalized linear models with robust variances to provide incidence rate ratios (IRRs) comparing the incidence of CPRD (GP) consultations, CPRD (GP) prescriptions, and HES (hospital) admissions following the first, second, and third bleeds to the incidence in the period prior to the first bleed. To estimate the impact of incremental bleeding events on health care costs, the GP consultation and prescription rates were annualized and direct costs estimated by applying a unitary cost from the NHS reference costs and the Personal Social Services Research Unit for primary care resource in the United Kingdom to each of the medical resources consumed. 20 21 Log-gamma generalized linear models were used to provide ratios comparing the GP consultation and prescription costs following the first, second and, third bleeds to the costs in the period prior to the first bleed. Similar to the Cox models, both unadjusted and adjusted models were estimated with the number of bleeds coded as a four-level time-varying categorical variable.

The main analysis assumed that each bleed code represented a separate bleeding event; this assumption was tested by carrying out sensitivity analyses in which bleed codes recorded within 1, 7, or 14 days after a previous bleed code were assumed to relate to the previous bleeding event. Sensitivity analyses were also used to investigate the sensitivity of the results to censoring follow-up at 3, 6, 12, and 24 months. A post-hoc sensitivity analysis including a random effect at the patient level to account for the correlation of failure times within individuals was also performed.

## Results

Among 184,599 patients in the database who started VKA treatment during the study period, we identified 29,489 eligible patients per inclusion criteria defined for this study ( Supplementary Fig. S1 ). The median follow-up time was 22 patient-months. Demographic and clinical characteristics are reported in Table 1 . The mean age was 73.4 (standard deviation [SD]: 10.5), 58% of patients were men, and 82% were Caucasian.

**Table 1:** Patient characteristics per bleed group (at the time of changing bleed exposure group)

	All CPRD-HES incident patients	1 bleed ( *n* = 4,308)	2 bleeds ( *n* = 1,512)	3 bleeds ( *n* = 555)
Age, mean (SD)	73.4 (10.5)	77.1 (8.9)	78.4 (8.7)	78.8 (8.6)
Median	75	78	79	79
Min–max	22–101	29–103	29–100	34–101
P10, p50, p90	60, 75, 85	65, 78, 88	67, 79, 89	67, 79, 90
**Age groups**				
≤39	0.6% (178)	0.2% (7)	0.3% (4)	0.2% (1)
40–50	2.5% (723)	0.7% (30)	0.5% (7)	0.0% (0)
51–60	8.2% (2,405)	3.3% (141)	2.5% (38)	2.3% (13)
61–70	22.9% (6,756)	16.6% (715)	13.2% (199)	13.3% (74)
71–80	39.2% (11,569)	41.4% (1,785)	40.1% (606)	39.5% (219)
≥81	26.6% (7,858)	37.8% (1,630)	43.5% (658)	44.7% (248)
**Gender**				
Men, % ( *n* )	57.8% (17,048)	57.6% (2,480)	57.5% (869)	60.2% (334)
Women, % ( *n* )	42.2% (12,441)	42.4% (1,828)	42.5% (643)	39.8% (221)
**BMI**				
*N*	14,165	2,296	829	316
Mean (std)	29.1 (6.3)	28.3 (5.9)	27.9 (6.1)	27.6 (5.9)
Median	28	27	27	27
Min–max	13–68	14–64	12–59	14–58
P10, p50, p90	22.2, 28.2, 37.2	21.6, 27.4, 36	21.1, 27, 35.4	21.3, 26.6, 35
Other or missing	52.0% (15,324)	46.7% (2,012)	45.2% (683)	43.1% (239)
**Smoking status, % (** ***n*** **)**				
Current	10.9% (3,229)	8.9% (384)	8.9% (135)	7.4% (41)
Past	29.1% (8,574)	33.7% (1,452)	33.7% (509)	33.2% (184)
Never	25.3% (7,455)	24.9% (1,074)	24.8% (375)	26.1% (145)
Missing	34.7% (10,231)	32.5% (1,398)	32.6% (493)	33.3% (185)
**Comorbidities**				
Alcoholism, % ( *n* )	3.7% (1,087)	2.8% (121)	2.9% (44)	3.1% (17)
Hypertension, % ( *n* )	35.0% (10,309)	44.5% (1,917)	51.4% (777)	53.0% (294)
Diabetes mellitus, % ( *n* )	10.7% (3,153)	14.0% (605)	16.1% (244)	18.4% (102)
Congestive heart failure, % ( *n* )	12.2% (3,606)	14.5% (625)	19.8% (299)	20.4% (113)
Peripheral artery disease, % ( *n* )	2.9% (863)	3.3% (142)	3.8% (58)	2.7% (15)
Liver disease, % ( *n* )	0.5% (140)	0.8% (36)	1.1% (17)	2.3% (13)
Malignancies, % ( *n* )	6.2% (1,838)	10.3% (443)	12.5% (189)	14.8% (82)
**HAS-BLED**				
Mean (SD)	2.0 (1.0)	2.5 (1.1)	2.5 (1.1)	2.5 (1.0)
P10, p50, p90	1, 2, 3	1, 2, 4	1, 2, 4	1, 2, 4
0	6.1% (1,809)	1.6% (68)	1.9% (28)	0.7% (4)
1	21.6% (6,366)	16.2% (697)	15.4% (233)	15.0% (83)
2	41.8% (12,321)	35.1% (1,510)	34.5% (522)	35.5% (197)
3	23.6% (6,973)	31.6% (1,363)	30.6% (462)	31.5% (175)
4	6.2% (1,842)	12.4% (533)	14.9% (225)	13.9% (77)
5	0.6% (169)	3.0% (131)	2.6% (40)	3.1% (17)
6	0.0% (9)	0.1% (6)	0.1% (2)	0.4% (2)
**CHA2DS2-VASc**				
Mean (SD)	2.7 (1.6)	3.0 (1.5)	3.2 (1.5)	3.2 (1.4)
P10, p50, p90	1, 3, 5	1, 3, 5	1, 3, 5	1, 3, 5
0	8.3% (2,437)	3.5% (149)	2.4% (36)	1.4% (8)
1	15.6% (4,599)	10.1% (436)	8.8% (133)	9.7% (54)
2	23.8% (7,029)	24.4% (1,049)	19.9% (301)	19.6% (109)
3	24.2% (7,150)	28.5% (1,228)	26.7% (404)	29.5% (164)
4	14.6% (4,319)	18.7% (807)	23.4% (354)	21.1% (117)
5	8.0% (2,368)	9.4% (407)	12.8% (194)	12.6% (70)
6	3.9% (1,147)	3.6% (155)	4.2% (63)	4.3% (24)
7	1.2% (354)	1.6% (68)	1.5% (23)	1.4% (8)
8	0.3% (75)	0.2% (8)	0.2% (3)	0.2% (1)
9	0.0% (11)	0.0% (1)	0.1% (1)	0.0% (0)
Aspirin prescription before index date, a % ( *n* )	59.9% (17,673)	31.1% (1,339)	27.1% (409)	24.0% (133)
Concomitant aspirin use, b % ( *n* )	32.7% (9,653)	22.4% (963)	25.9% (391)	27.6% (153)

Abbreviations: CPRD-HES, Clinical Practice Research Datalink—Hospital Episode Statistics; SD, standard deviation; VKA, vitamin K antagonist.

a Index date was the date of VKA initiation.

b This variable was calculated based on aspirin prescriptions occurring within 90 days after VKA initiation (includes date of VKA initiation). This variable captures if there was at least 1 day of overlap between an aspirin and a VKA prescription (accounting for treatment durations).

Overall, 14.6% ( *n* = 4,308) of the population experienced at least one AB event following initiation of VKA treatment. Among individuals who continued on VKA after their first AB event, 35.3% ( *n* = 1,512) experienced at least one subsequent AB event. The proportion of individuals experiencing an AB event after a second and third bleed was marginally higher: at 37 and 40.3%, respectively ( Supplementary Table S1 ). In adjusted Cox analyses, we observed a fourfold increased risk of a recurrent AB event following a first bleed (HR: 4.2; 3.9–4.5) and a fivefold to eightfold increased risk following a second (HR: 5.6; 5.0–6.2) and third (HR: 8.4; 7.4–9.6) AB event compared with follow-up time without AB ( Table 2 ). The HRs observed are suggestive of an incremental effect of the number of bleeds on the risk of bleeds.

**Table 2:** Unadjusted and adjusted risks of subsequent bleeds (AB variable) expressed by hazard ratios obtained from time-varying Cox models

	HR _unadj_ (CI _95_ )	HR _adj_ (CI _95_ ) a
0 bleeds (reference group)	–	–
1 bleed	4.21 (3.94–4.50)	4.17 (3.90–4.47)
2 bleeds	5.75 (5.16–6.40)	5.57 (5.00–6.20)
≥3 bleeds	8.66 (7.56–9.92)	8.42 (7.39–9.60)

Abbreviation: AB, any bleed; CI, confidence interval; HR, hazard ratio.

a Adjusted for variables listed in Table 1 .

**Fig. 2:**
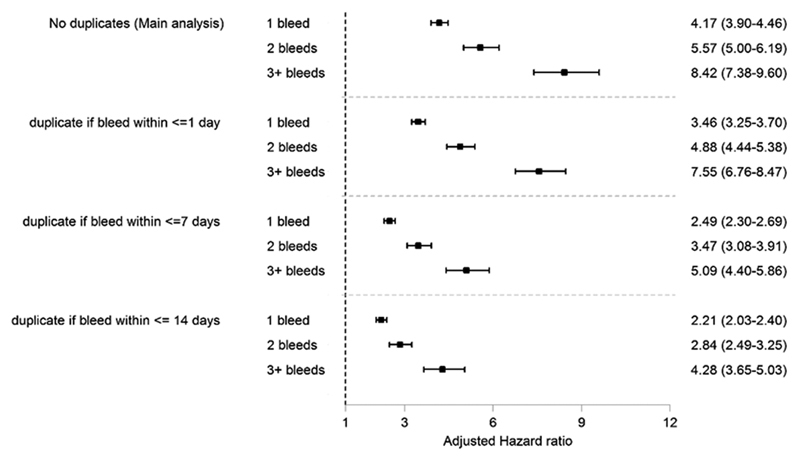
Results of sensitivity analysis investigating the impact of treating records of bleeds as duplicates if they are recorded within certain time periods of each other. The main analysis treats all records of bleeds as separate events of bleeds. Hazard ratios adjusted for all characteristics listed in Table 1 .

The results of sensitivity analyses for the primary outcome (AB) were sensitive to assuming AB codes recorded within close proximity relate to the same AB event ( Fig. 2 ) and to censoring at fixed time periods ( Supplementary Fig. S2 ). Sensitivity analyses included a random effect at the patient level also provided lower adjusted HRs: 2.94 (CI _95_ : 2.75–3.14), 2.51 (CI _95_ : 2.26–2.77), and 2.06 (CI _95_ : 1.82–2.34) for the period following the first, second, and third bleeds, respectively, and suggested no incremental increase in the hazard of bleeding with increasing number of bleeds. Across all AB analyses, the risk of bleeding was elevated at least twofold in all periods following the first bleed.

**Fig. 3:**
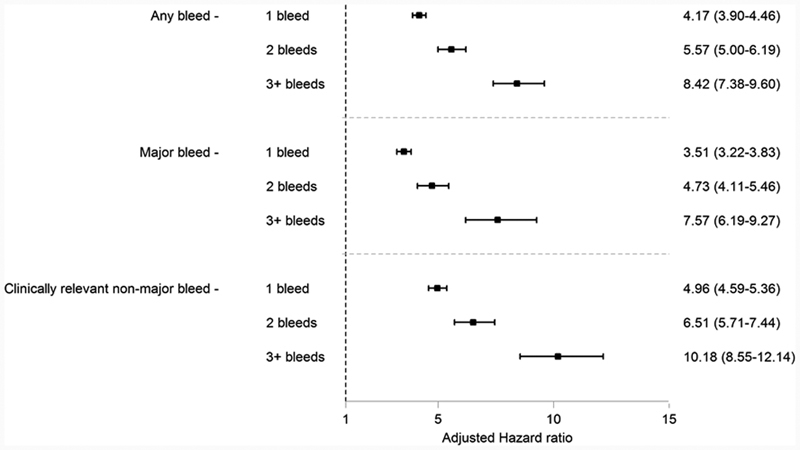
Forest plot describing the hazard of primary and secondary bleed outcomes during VKA treatment in the period following the first, second, and third bleeds compared with the hazard in the period before any bleeds have occurred. Hazard ratios adjusted for all characteristics are listed in Table 1 . VKA, vitamin K antagonist.

Considering both MB and CRNMB separately, 8.1% of the population had at least one MB event while 6.5% had at least one CRNMB. The proportions having CRNMB events after the first, second, and third bleeds were higher than the proportions having MB events in the same period ( Supplementary Table S1 ), resulting in slightly higher adjusted HRs for CRNMB than MB in each period ( Fig. 3 ). The hazard of ICBs was also higher after the first (HR: 4.18 [CI _95_ : 3.35–5.23]), second (HR: 8.94 [CI _95_ : 6.63–12.07]), and third bleeds (HR: 13.15 [CI _95_ : 7.69–22.48]) relative to the hazard prior to the first bleed.

**Fig. 4:**
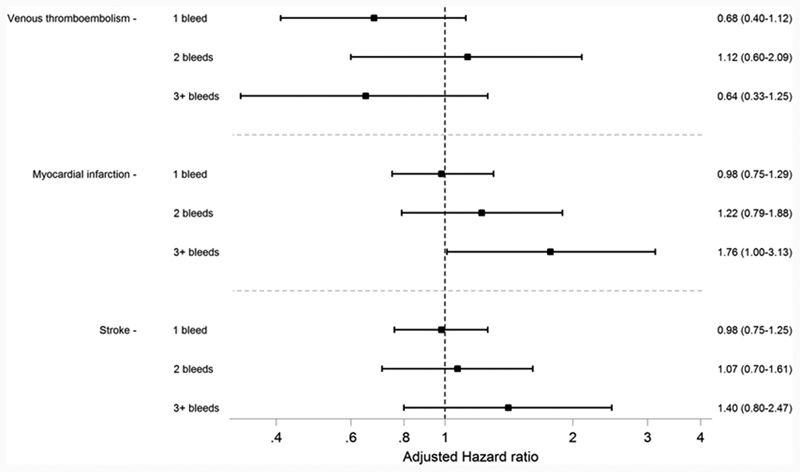
Forest plot describing the hazard of secondary clinical outcomes during VKA treatment in the period following the first, second, and third bleeds compared with the hazard in the period before any bleeds have occurred. Hazard ratios adjusted for all characteristics listed in Table 1 . VKA, vitamin K antagonist.

The proportions of individuals in the study population experiencing VTE (2.3%), MI (2.3%), or stroke (2.9%) after initiating VKA were lower than the proportions experiencing bleeding events ( Supplementary Table S1 ). Adjusted HRs suggest that the number of bleeding events had no impact on the number of VTE, MI, or stroke events observed with all CIs crossing the line of unity (one) ( Fig. 4 ).

**Fig. 5:**
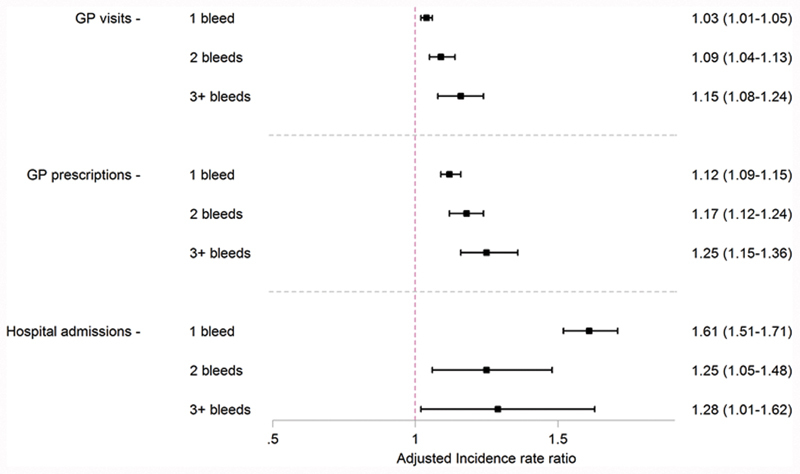
Forest plot describing the incidence rate of GP visits, GP prescriptions, and hospital admissions during VKA treatment in the period following the first, second, and third bleeds compared with the incidence in the period before any bleeds have occurred. Incidence rate ratios adjusted for all characteristics listed in Table 1 . GP, general practitioner; VKA, vitamin K antagonist.

On average, individuals in the study population had 2.6 GP visits per month (SD: 2.0), 7.0 GP prescriptions per month (SD: 7.1), and 0.1 hospitalizations per month (SD: 0.4). The mean number of GP visits, GP prescriptions, and hospitalizations per month after the first, second, and third bleeds were 3.9, 3.0, and 3.0 GP visits, 7.2, 7.2, and 8.2 GP prescriptions per month, and 2.9, 1.8, and 0.7 hospitalizations per month, respectively. Adjusted IRRs suggest that, after accounting for differences in measured patient characteristics, the incidence of all three HCRU outcomes after initiating VKA treatment was higher following the first, second, and third bleeds than before ABs had occurred ( Fig. 5 ). There is little evidence of an incremental effect for any of the HCRU outcomes.

The impact of bleeding events on HCRU was also quantified in terms of the impact on costs associated with GP visits and GP prescriptions. Total costs associated with GP visits were observed to be 72, 52, and 24% higher following the first, second, and third bleeds than in the period prior to the first bleed ( Supplementary Fig. S3 ). The costs associated with prescriptions were observed to be 25 to 30% higher after the first, second, and third bleeds ( Supplementary Fig. S3 ).

## Discussion

In this population-based, real-world study, the risk of clinically relevant bleeding events in individuals being treated with a VKA for NVAF was observed to be elevated at least twofold following the first clinically relevant bleeding event. In contrast, the risk of VTE, stroke, and MI was not found to differ according to the number of clinically relevant bleeding events. The rate of GP consultations, GP prescriptions, and hospitalizations as well as the cost of GP consultations and GP prescriptions was increased in all periods relative to the prebleed period but was not found to increase incrementally with each subsequent bleed.

The data source used in this study provides real-world data for a large, relatively unselected sample of the population of England. Given the observational nature of the data, there are several sources of systematic error that must be considered when interpreting the results of specific studies. Prescriptions issued outside primary care are not captured in the CPRD-HES-linked dataset; in an effort to account for this, we have used INR records as markers of VKA prescriptions and have assumed that VKA treatment is continuous in the presence of gaps in treatment of up to 45 days. Despite this, if an individual received more than 45 days VKA supply outside primary care and had their INR recorded outside primary care during this time, we may inappropriately assume that they discontinued therapy and censor them. As a result, our findings may not be generalizable to patients whose VKA treatment is managed outside primary care for extended periods of time. Our results relate to clinically relevant bleeding only, as bleeds that are not recorded during GP consultations and hospitalizations will not be identified in the study. As we are investigating recurrent bleeds there is a possibility that some bleed records may represent re-recording of an earlier event in the same individual; sensitivity analyses suggested that if this were the case the main analysis will have overestimated the relative differences in the hazard of bleeding between pre- and postbleed periods. As VTE, stroke, and MI were not included as recurrent events, duplicate records are not an issue; however, some false-positive misclassification is possible if, for example, incorrect working diagnoses are recorded in the primary care record; however, the validity of diagnoses recorded in the CPRD is reported to be high. 16 Despite including a considerable number of covariates in the multivariate analyses, misclassification of measured covariates or a lack of data on other potential confounders may have resulted in residual confounding. For example, it was not possible to capture labile INR or concomitant use of drugs that increase the risk of bleeding for inclusion in the calculation of HAS-BLED scores; as a result, we may not have full control for differences in bleeding risk factors.

In the main analysis the risk of clinically relevant bleeding events increased incrementally according to the number of prior bleeds, from four times the risk following the first bleed to eight times the risk following the third or more bleeds. However, two of the three sensitivity analyses that were performed provided strong evidence to question these findings. The first of these sensitivity analyses explored the possibility that GPs entered multiple codes to record a single bleeding event, for example, at initial presentation and at a follow-up visit 2 weeks later. The second sensitivity analysis accounting for this grouped bleeding events if they occurred within increasing time periods of each other and found that as the grouping period was increased the HRs that were observed decreased. This suggests that the sizable HRs in the main analysis may be due in part to data-quality issues rather than a biological mechanism. The second sensitivity analyses explored the possibility that the results observed in the main analysis were partly due to unobserved heterogeneity between patients. In addition to suggesting that the HRs were lower than those in the main analysis, this sensitivity analysis also suggested that there was no incremental increase in hazard compared with that observed following the first bleed. Despite both of these sensitivity analyses raising questions regarding the robustness of the results of the main analyses, it is notable that across all analyses the risk of clinically relevant bleeding events was increased at least twofold in the period following the first and each subsequent bleed, relative to the period prior to the first bleed.

In England, the National Institute for Health and Care Excellence (NICE) recommends that among individuals with AF and CHA2DS2-VASc scores of 1 (in men) or 2 (in women), OAC treatment should be considered but that bleeding risk should be taken into account in such treatment decisions. 22 As with many other guidelines, NICE recommends bleeding risk in individuals diagnosed with AF be assessed using the HAS-BLED score. 22 As history of major bleeding is captured in the HAS-BLED score, the increased risk associated with previous bleeds is likely taken into account in current treatment decisions. 19 In agreement, the consensus summary published by the European Society of Cardiology Working Group on Thrombosis suggests that after bleeding, OAC should be reinitiated as soon as the cardiovascular thrombotic risks associated with discontinuation are thought to outweigh the risk of rebleeding with reinitiation (in most cases within 1 week) as long as the bleeding event is not a life-threatening intracranial or extracranial bleed. 23 Meta-analyses have demonstrated that individuals who restart OAC treatment following a bleed have a net clinical benefit compared with those who do not restart treatment. This net clinical benefit arises as the increase in the risk of bleeding is offset by a reduction in the risk of thromboembolic events and death. 24 25 As a result, treatment restart is considered warranted in many clinical situations, particularly among individuals with less severe bleeds and with reversible causes of bleeding.

Despite efforts to control for confounding, the studies contributing to these meta-analyses are likely limited by their ability to account for confounding by indication: the fact that OAC treatment is more likely to be restarted in individuals who have less severe bleeds with reversible causes, and who may therefore be at an inherently lower risk of adverse outcomes, such as thromboembolic events and death, than those who restart OACs. In the present study, rather than comparing those who restart OAC treatment to those who do not restart, we compared the risk of bleeding over time within a population of individuals who were all restarted on OACs. While the present study is therefore potentially limited by unmeasured time-varying confounding, it is less susceptible to confounding by indication. An added limitation of this study relative to some previous studies is the lack of data on mortality.

While the literature suggests that restarting VKAs postbleed is preferable to discontinuation of anticoagulant therapy, this does not reflect the current clinical decision problem as VKAs are no longer the only available treatment option. NOACs represent an appealing treatment option in the postbleed setting due to their lower risk of bleeding compared with VKAs. 11 Due to the calendar period included in our study, it was not possible to explore whether individuals using NOACs experienced increases in the risk of bleeding following their first bleed, similar to those observed with VKAs. An ongoing phase II trial of apixaban versus discontinuation of OAC treatment in the postbleed setting will begin to provide the evidence needed to inform treatment decisions. 26 However, studies providing head-to-head comparisons of VKAs and NOACs would provide the optimum evidence on which to base treatment decisions in the postbleed setting.

In conclusion, the doubling in the risk of bleeding following the first bleed, taken alongside the stable risk of VTE, MI, and stroke observed over the same periods, suggests that among individuals being treated with VKAs the risk–benefit balance for VKA treatment should be reconsidered following the first bleed. Further work is needed to inform decisions regarding alternative treatment options in the postbleed setting.
