# Supplementary material for: Risk of Recurrent Bleeding Events in Nonvalvular Atrial Fibrillation Treated with Vitamin K Antagonists: A Clinical Practice Research Datalink Study
Source: TH Open. 2019 Oct 4;3(4):e316–24. doi: 10.1055/s-0039-1698413 (PMC6777986; doi:10.1055/s-0039-1698413)
Supplement: Supplementary file 1 — Supplementary Material [file 10-1055-s-0039-1698413-s190001.pdf]

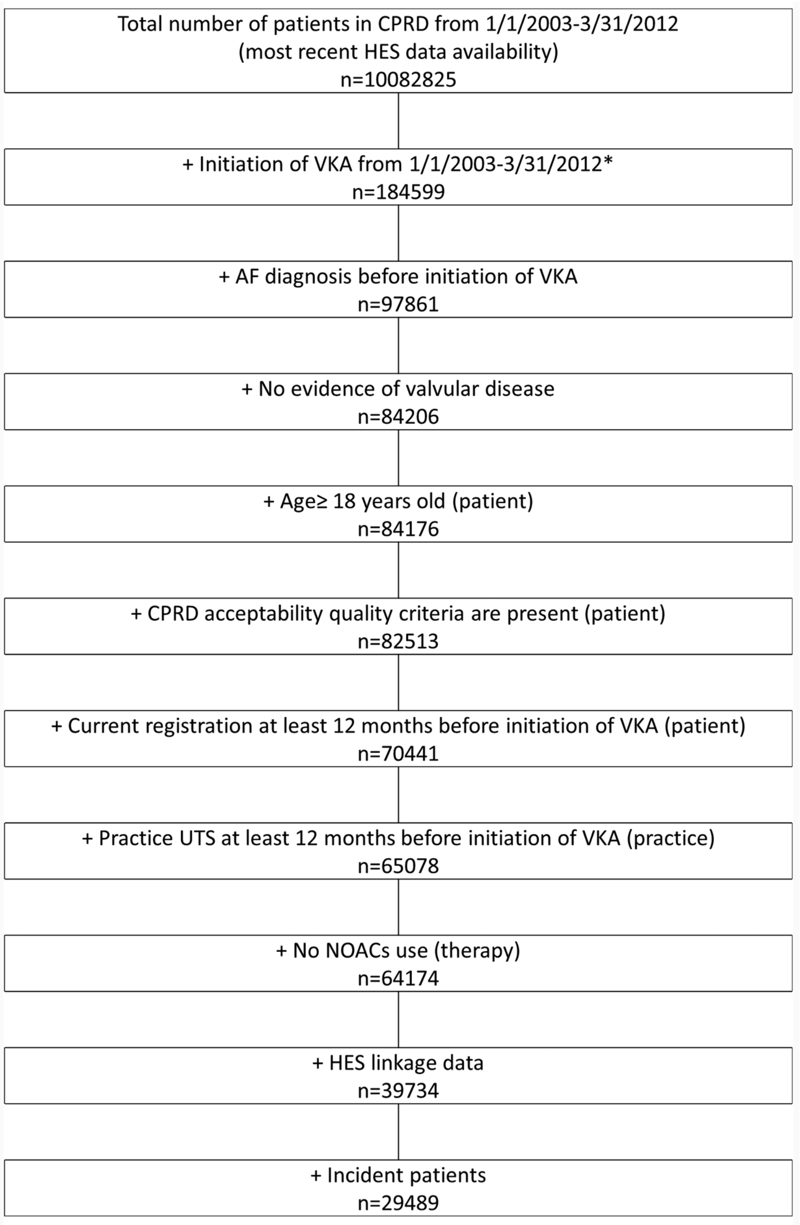

Supplementary Fig. S1 Flow diagram describing derivation of study population.

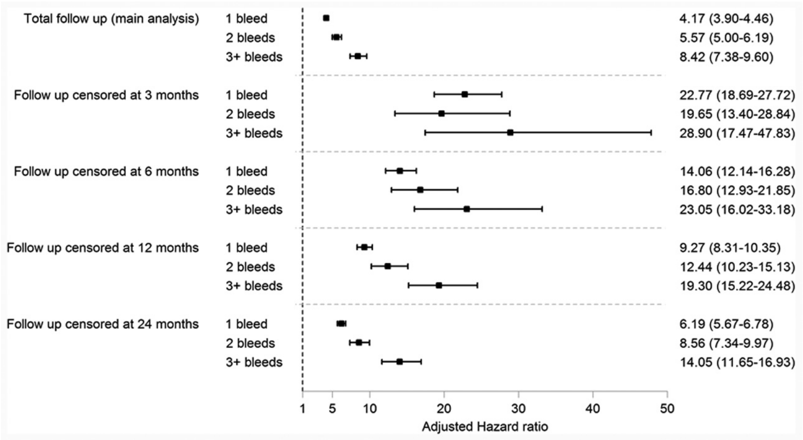

Supplementary Fig. S2 Results of sensitivity analysis investigating the impact of censoring follow-up at 3, 6, 12, and 24 months. Hazard ratios adjusted for all characteristics listed in ►**Table 1**.

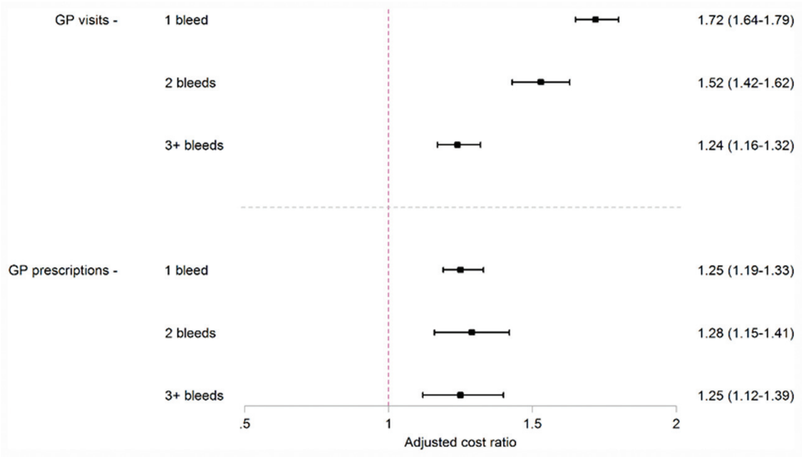

Supplementary Fig. S3 Forest plot describing the cost of GP consultations and GP prescriptions during VKA treatment in the period following the first, second, and third bleeds compared with the cost in the period before any bleeds have occurred. Cost rate ratios adjusted for all characteristics listed in ►**Table 1**. GP, general practitioner; VKA, vitamin K antagonist.

Supplementary Table S1 Number of events observed in the entire study population and within each of the three exposure periods

	All patients ($n = 29,489$)	1 bleed ($n = 4,308$)	2 bleeds ($n = 1,512$)	3+ bleeds ($n = 555$)
Outcome	*N* (%)	*N* (%)	*N* (%)	*N* (%)
Any bleeding	4,308 (14.6%)	1,512 (35.3%)	555 (37%)	221 (40.3%)
Major bleeding	2,393 (8.1%)	692 (16.2%)	249 (16.6%)	112 (20.4%)
CRNMB	1,915 (6.5%)	820 (19.2%)	306 (20.4%)	134 (24.4%)
VTE	689 (2.3%)	42 (1.0%)	25 (1.7%)	10 (1.8%)
Myocardial infarction	672 (2.3%)	59 (1.4%)	21 (1.4%)	13 (2.4%)
Stroke	867 (2.9%)	73 (1.7%)	24 (1.6%)	13 (2.4%)

Abbreviations: CRNMB, clinically relevant nonmajor bleeding; VTE, venous thromboembolism.
